# Safety and efficacy of vemurafenib in BRAF V600E mutation-positive metastatic melanomas

**DOI:** 10.1186/1479-5876-13-S1-P7

**Published:** 2015-01-15

**Authors:** Andrea P Sponghini, David Rondonotti, Marco Giavarra, Roberto Giorgione, Francesca Platini

**Affiliations:** 1 AOU Maggiore della Carità, S.C. di Oncologia, Novara, Italy; 2 AOU Maggiore della Carità, S.C. di Dermatologia, Novara, Italy

## Background

Metastatic melanoma has a poor prognosis, and 5-year survival is 65% with regional stage disease and 15% with distant stage disease. Chemotherapy has limited success in metastatic melanoma, with a median overall survival of 8 months. Malignant melanoma is not a singular, homogeneous disease but rather a mixture of subtypes characterized by specific mutations. Tumours with BRAF mutations respond to BRAF kinase inhibitor vemurafenib, that was approved by US FDA in 2011 and EMA in 2012 for therapy of patients with advanced melanoma, harboring mutation in BRAF V600E gene. Some randomized clinical trials focused on the significant reduction in the risk of death and disease progression associated with vemurafenib, compared with classical standard chemotherapy. Vemurafenib is generally well tolerated with the most common side effects being arthralgia, photosensitivity, fatigue and dermatitis.

## Materials and methods

From January 2013 to August 2014 we have collected data of 16 patients with metastatic melanoma (IV stage). Metastatic sides were: brain (35%), liver (29%), skin (24%) and lung (22%). We estimated overall survival (OS) and toxicities therapy-related. Patients were eligible if their tumour tissue was positive for the presence of BRAF V600E mutation. Dosing of vemurafenib ranged from 480 mg/bid to 960 mg/bid. Each patient has been revalued every two weeks with clinical examination until conclusion of treatment. Treatment was discontinued on disease progression or toxicity.

## Results

Median overall survival was 13 months. The most common adverse effects included arthralgia (40%), fatigue (35%) and photosensitivity reactions (25%), grade 1 or 2 side effects as per the Common Terminology Criteria for Adverse Events (CTCAE). Elevated liver enzymes were documented in close to 10% of treated patients and no prolongation of the QTc interval, cardiac arrhythmias, keratoacanthoma and squamous cell carcinoma were reported. Dosing’s modification has been required in three patients: two patients have discontinued the treatment and they have been resumed at 720 mg/bid while one patient have been resumed at 480 mg/bid. Nobody needed to stop the treatment due to unacceptable toxicities.

## Conclusions

According to the findings available in literature, inhibition of BRAF improves clinical outcome in patients with the BRAF V600E mutation. Vemurafenib was well tolerated and adverse event profiles were similar to those reported in literature.

